# A Case Report of Immune Checkpoint Inhibitor-Induced Aortitis Treated with Tocilizumab

**DOI:** 10.1155/2022/7971169

**Published:** 2022-10-12

**Authors:** Chance H. Bloomer, Rahul V. Annabathula, Vanya Aggarwal, Bharathi Upadhya, Thomas W. Lycan

**Affiliations:** Internal Medicine, Atrium Health at Wake Forest Baptist, Winston Salem, NC, USA

## Abstract

Vasculitic immune checkpoint inhibitor-related adverse events (irAEs) are rare, with limited data to guide their management. Here, we present a case of a 67-year-old female with stage IV cutaneous melanoma who received first-line pembrolizumab. She had completed 21 cycles of pembrolizumab dosed at 200 mg every 21 days over 15 months when she developed fatigue, chills, decreased appetite, night sweats, nausea, diarrhea, dry cough, and chest pain. A routine, staging positron emission tomography (PET) scan revealed aortitis of the transverse aortic arch. An extensive workup was unremarkable for other causes, so her condition was labeled a grade III immune-related vasculitis. Based on this diagnosis, we started high-dose prednisone and discontinued pembrolizumab. After two months of high-dose prednisone, she developed bothersome weight gain and insomnia, leading to a switch from prednisone to tocilizumab as a steroid-sparing agent. The selection of tocilizumab was based on its routine use for giant cell arteritis which can have extracranial symptoms including thoracic aortitis. Her symptoms resolved, and subsequent PET scans showed resolution of the aortitis and no evidence of metastatic melanoma. As the indications for immunotherapy expand, rare complications are becoming more prevalent, and more data will be needed to guide their management. While there is evidence for tocilizumab use as a steroid-sparing treatment for large-vessel vasculitides due to other conditions, this is the first case of its use to treat an aortitis irAE to our knowledge. In this case, it was an effective means of treating the patient while sparing them from prolonged corticosteroids.

## 1. Introduction

Immune checkpoint inhibitors are a promising new class of cancer treatments that inhibit the ability of cancer cells to hide from cytotoxic immune cells. Since their first regulatory approval in 2011, these drugs have become a standard-of-care treatment option for an estimated 40% of all patients with cancer in the United States [1]. These drugs have fewer side effects than cytotoxic chemotherapy butcan causeimmune-related adverse events (irAEs) that are less predictable and can affect any organ system. One rare but severe irAE is aortitis, which has not been reported in any major treatment trials to date but has been described in case reports [2, 3]. Optimal management is uncertain; most of these case reports were treated similarly to small vessel vasculitic irAEs. In general, professional guidelines recommend systemic corticosteroids as initial treatment followed by infliximab, rituximab, or tocilizumab for refractory cases [4–6]. These guidelines have variable strength of evidence to support the different recommendations, ranging from clinical trials to expert opinion. Notably, there has been no published patient data to guide the use of tocilizumab to treat an aortitis irAE such as the one we present in this case report [4–6].

Tocilizumab is an IL-6 inhibitor that prevents the activation of a variety of proinflammatory and immune functions by preventing the binding of IL-6 to its receptor. Because of its broad effects on the immune system, it is used in various inflammatory mediated diseases such as cytokine release syndrome, rheumatoid arthritis, and vasculitis [7].

## 2. Case Report/Case Presentation

Our case is of a 67-year-old female initially diagnosed with stage IV cutaneous melanoma with metastases to the brain (*n* = 21) as well as to the mesenteric, inguinal, and external iliac lymph nodes. She was treated with whole-brain radiation therapy followed by first-line pembrolizumab dosed at 200 mg every 21 days.

**Figure 1:**
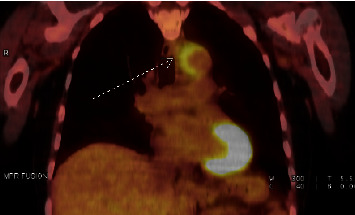
Initial positron emission tomography scan showing aortitis. Increased metabolic activity of the wall of the transverse aortic arch (arrow) and surrounding inflammation were suggestive of aortitis. Imaging was otherwise notable for a complete response with no evidence of metastatic disease.

After 15 months of treatment, she had gradual new onset of fatigue, chills, decreased appetite, night sweats, nausea, diarrhea, dry cough, and atypical chest pain over three days. She visited an urgent care clinic twice for her symptoms. She received symptomatic treatment including acetaminophen, ibuprofen, a 10-day course of amoxicillin/clavulanic acid for possible otitis media, loperamide, and IV fluids. Her symptoms resolved and she was symptom-free by the time she saw her oncologist one week later. She had a routine surveillance PET/CT (Figure 1) that showed increased metabolic activity in the wall of the transverse aortic arch and mediastinal fat stranding around the area indicative of inflammation that together suggested aortitis due to unclear etiology. Imaging also showed a complete response to treatment with no evidence of metastatic disease.

The main concern was that the aortitis was an irAE from pembrolizumab, and because of the potentially life-threatening nature of her condition, she was empirically started on prednisone at 1 mg/kg/day while other possible causes were worked up. Labs done to rule out other infectious or autoimmune causes included complete blood count, comprehensive metabolic panel, two sets of blood cultures, influenza A/B, Clostridium difficile, hepatitis panel, rapid plasma reagent, urinalysis, urine culture, sedimentation rate, antinuclear antibodies, rheumatoid factor, C3, C4, c-ANCA, p-ANCA, and C-reactive protein with the only significant result being an *E. coli* urinary tract infection. Once the complete workup returned as unremarkable, the symptoms were attributed to an irAE from pembrolizumab. Due to the danger associated with taking biopsies of large vessels, the diagnosis was not confirmed histologically, but the clinical context and response to immunosuppression were strongly supportive of this diagnosis.

The patient was started on high-dose corticosteroids and was referred to rheumatology, which recommended starting tocilizumab and beginning a slow corticosteroid taper to avoid a prolonged corticosteroid course and minimize insomnia and weight gain which she was experiencing from her current corticosteroid dose. Repeated levels of inflammatory markers done after the start of steroids remained within normal limits. When her taper was started, she had already been taking 80 mg daily of prednisone for two weeks, so she had decreased to 70 mg daily for two weeks, 60 mg daily for two months, 50 mg for two weeks, 40 mg for five weeks, then 10 mg decreases every two weeks until her last dose of 5 mg daily for two weeks. Her 60 mg dosing period was prolonged as it was viewed as the treatment dose rather than the 80 mg dose to lessen the side effects. Her 40 mg dosing period was prolonged as they allowed her to start the tocilizumab and ensure tolerance prior to tapering further. In total, her corticosteroid regimen lasted approximately six months.

**Figure 2:**
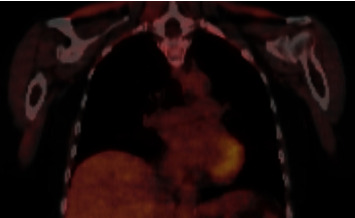
Positron emission tomography scan showing resolution of aortitis. This PET scan was done approximately three months after beginning prednisone and did not show any of the previous areas of increased metabolic activity or inflammation. It also did not show any evidence of recurrence of her metastatic melanoma.

Three months after diagnosis, a follow-up PET scan (Figure 2) showed resolution of the aortitis and tocilizumab 162 mg IV weekly was started as a steroid-sparing agent. She received tocilizumab for ten months and tolerated it well with no significant side effects until it was stopped once there was a sustained complete response. Her disease was considered resolved at this time based on a combination of symptom resolution of repeated PET scans that showed no inflammation. Pembrolizumab was permanently discontinued after initial aortitis diagnosis. At last known follow-up three years after the irAE, quality of life was good with follow-up PET/CT scans that remained negative for recurrent aortitis or metastases.

## 3. Discussion/Conclusion

To date, there are only a handful of care reports describing aortitis as an irAE, and it is usually discussed along with other vasculitis irAEs or irAEs in general [2]. The guidelines of several major medical societies including the American Society of Clinical Oncology (ASCO), National Comprehensive Cancer Network (NCCN), and Society for Immunotherapy of Cancer discuss treatment strategies for vasculitis irAEs, and the ASCO and NCCN discuss tocilizumab specifically as a treatment option for refractory vasculitis [4–6]. However, we were unable to find any previous examples of tocilizumab being used as treatment for an aortitis irAE. Therefore, the decision to use it was based on a combination of these guidelines as well as from the treatment of giant cell arteritis (GCA), the most common large vessel vasculitis which is often associated with thoracic aortitis [8]. There was an additional degree of uncertainty in that we were never able to confirm the diagnosis via biopsy for the reasons discussed above, but her response to the treatment was reassuring that she was treated appropriately.

Although there are no available examples of tocilizumab to treat an aortitis irAE, two literature reviews about its use to treat other vasculitic irAEs have shown encouraging results. In total, the two studies included 113 patients and 87% (*n* = 89) of those who had their irAE outcomes reported (*n* = 102) showed clinical improvement after the treatment [9, 10]. One of the included cases was of large vessel vasculitis of the left common carotid, right subclavian, and brachiocephalic trunk and this patient was successfully treated [9].

In conclusion, our case highlights the clinical presentation of an aortitis irAE and the importance of maintaining a high clinical suspicion for it and other potentially life-threatening irAEs. Had our patient not undergone a routine PET/CT, she likely would not has been diagnosed until her symptoms had recurred and possibly worsened. This is also the first documented case of tocilizumab to treat an aortitis irAE to our knowledge and shows that it can be used to successfully treat such a condition while avoiding prolonged corticosteroid courses. Although its use is supported by clinical guidelines, additional data are needed to better evaluate precise treatment regimens and indications.

## Abbreviations

irAEs: Inhibitor-related adverse events
PET: Positron emission tomography
GCA: Giant cell arteritis
NCCN: National comprehensive cancer network
ASCO: American society of clinical oncology.
